# Processing and Nutritional Quality of Breakfast Cereals Sold in Italy: Results from the Food Labelling of Italian Products (FLIP) Study

**DOI:** 10.3390/nu15082013

**Published:** 2023-04-21

**Authors:** Donato Angelino, Monica Dinu, Barbara Gandossi, Nicoletta Pellegrini, Daniela Martini

**Affiliations:** 1 Department of Bioscience and Technology for Food, Agriculture and Environment, University of Teramo, 64100 Teramo, Italy; 2 Department of Experimental and Clinical Medicine, University of Florence, 50134 Florence, Italy; 3 Department of Food, Environmental and Nutritional Sciences (DeFENS), University of Milan, 20133 Milan, Italy; 4 Department of Agricultural, Food, Environmental and Animal Sciences, University of Udine, 33100 Udine, Italy

**Keywords:** food label, NOVA system, ultra-processed foods, front-of-pack labelling

## Abstract

This study aimed to compare the level of processing (as assessed by the NOVA classification) and the nutritional quality (as assessed by nutrition values, Nutri-Score and NutrInform battery) of breakfast cereals currently on the Italian market. A total of 349 items were found, mostly belonging to the NOVA 4 group (66.5%) and to Nutri-Score C and A (40% and 30%, respectively). The NOVA 4 products showed the highest energy, total fat, saturates, and sugar content per 100 g and had the highest number of items with Nutri-Score C (49%) and D (22%). Conversely, NOVA 1 products had the highest content of fibre and protein, the lowest amounts of sugars and salt, and 82% of them were Nutri-Score A, while few Nutri-Score B and C were found. Differences were attenuated when products were compared for their NutrInform battery, with NOVA 4 items showing only slightly fuller batteries for saturated fats, sugar, and salt than NOVA 1 and NOVA 3 products. Overall, these results suggest that the NOVA classification partially overlaps with systems based on the nutritional quality of foods. The lower nutritional quality of NOVA 4 foods may at least partially explain the association found between the consumption of ultra-processed foods and the risk of chronic diseases.

## 1. Introduction

In recent years, increasing attention has been paid to the level of food processing [1]. NOVA is a classification system that groups foods according to the nature, extent, and purpose of the industrial processes they undergo, rather than in terms of nutrients [2]. In the NOVA classification, foods are assigned to one of the following four groups: NOVA 1 contains unprocessed or minimally processed foods, i.e., the edible parts of plants or animals taken directly from nature or minimally modified/preserved; NOVA 2 contains processed culinary ingredients, such as salt, sugar, oil, or starch, produced from NOVA 1 foods; NOVA 3 contains processed foods such as canned vegetables or freshly baked bread, produced by combining NOVA 1 and NOVA 2 foods; NOVA 4 contains ultra-processed foods, i.e., industrially formulated ready-to-eat products that are predominantly or entirely composed of food-derived substances and additives, with few or no intact foods from NOVA 1.

Up to date, many studies have shown an association between the consumption of NOVA 4 foods and health status, particularly regarding body weight [3], mortality [4], and chronic non-communicable diseases such as cardiovascular disease and depression [5,6]. Among the mechanisms hypothesized to explain these associations is that higher consumption of NOVA 4 foods leads to diets high in calories, free sugars, fat, and salt and low in dietary fibre [7]. Unlike the NOVA system, front-of-pack nutrition labels (FOPNLs) distinguish foods and beverages according to their energy and nutrient contribution to the overall diet. There are currently more than thirty different FOPNLs (proposed or implemented) in the world, many of them in use in multiple countries [8]. Some FOPNLs express the overall nutritional value of a food by using some or all the information from the nutrition declaration and/or other nutritional elements (e.g., the Nutri-Score, a graphic scale that divides the nutritional score into five classes expressed by a color and a letter). Other FOPNLs repeat specific numerical information from the mandatory nutrition declaration in a neutral manner (e.g., the NutrInform battery proposed by Italy).

Given that NOVA classification is not based on nutrient content, it is interesting to understand whether the classification of a food in NOVA 4 coincides with a worse classification by FOPNLs. In theory, NOVA and FOPNLs are complementary systems that focus on different aspects, so their application to a specific food may not necessarily lead to the same conclusions. An example is plant substitutes for animal foods that have a Nutri-Score A (indicating high nutritional quality) while being NOVA 4 [9]. This kind of discrepancy also emerged for other food groups, especially in a survey of foods on the Spanish market where only 75.5% of NOVA 4 foods were classified as having medium-low nutritional quality (C, D, and E) in the Nutri-Score [10]. Since there are no such comparisons made on products found in the Italian market, our aim was to compare the processing (as assessed by the NOVA classification) and the nutritional quality (as assessed by nutrition values retrieved in the nutritional declaration, Nutri-Score and NutrInform battery) of breakfast cereals currently on the Italian market, using data from the Food Labelling of Italian Products (FLIP) database. The choice of breakfast cereals comes from the fact that they can belong to different NOVA groups based on whether they are cereal-only, have added sugar or salt, or have many other ingredients not typically used domestically.

## 2. Materials and Methods

### 2.1. Data Collection and Extraction

Breakfast cereals included in the present work were selected as described in a previous study performed within the FLIP project [11]. The online search for the information was performed in July 2022.

The information retrieved for each product was the same as previously collected in a previous study [11], while the NOVA group was assigned to each item considering the processing classification system based on the NOVA classification [2,12].

The Nutri-Score was calculated for each item in accordance with the rules reported in the specific document (https://www.santepubliquefrance.fr/media/files/02-determinants-de-sante/nutrition-et-activite-physique/nutri-score/qr-scientifique-technique-en, Last access 1 February 2023).

Data for NutrInform battery (i.e., energy, fat, saturates, sugar, and salt content) were calculated by considering the standard serving size of 30 g [13] as defined in the manual of use (https://www.nutrinformbattery.it/Manuale_uso_NutrInform_Battery.pdf, Last access 1 February 2023).

Two researchers (DM and MD) extracted the data and double-checked the accuracy of data extraction, while inaccuracies were solved by a third researcher (DA).

All the retrieved data were collected in a Microsoft Excel database and sub-grouped for specific comparisons, i.e., tertiles of sugar, fibre, and salt, according to the NOVA group. Items were classified on the basis of the descriptive name as follows: (i) muesli, (ii) flakes, (iii) bran cereals, (iv) puffed cereals, and (v) others (e.g., cereals with honey or cream-filled cereals). Conversely, based on the presence of wholegrain ingredients, items were classified into refined, partially wholegrain (i.e., at least one ingredient), and wholegrain.

### 2.2. Data Analysis

Data were organized and statistically analyzed by using the Statistical Package for Social Sciences software (IBM SPSS Statistics, Version 28.0, IBM Corp., Chicago, IL, USA). The significance level was set at *p* < 0.05. Data of energy, nutrient, and fibre contents were expressed as median (interquartile range). In the descriptive analysis of the number of items, variables were expressed as absolute values. The Kolmogorov–Smirnov test was used to assess the normality of data distribution that was rejected.

Data were analyzed by means of the Mann–Whitney non-parametric test to allow comparisons of two independent groups or the Kruskal–Wallis test for independent samples with multiple pairwise comparisons. In addition, the variability of the nutritional values—as energy, nutrient, and fibre contents per 100 g of products—across the different items was assessed by means of a Principal Component (PC) analysis with varimax rotation, also considering the Nutri-Score and the NOVA group categorizations.

## 3. Results

### 3.1. Number and Characteristics of Food Items

Table 1 reports the number and the main characteristics of the retrieved items classified on the basis of the three NOVA groups (i.e., NOVA 1, 3, and 4). No items were classified as NOVA 2 since this group includes culinary ingredients. A total of 349 single items of breakfast cereals were included in the final evaluation, mostly belonging to the NOVA 4 group (66.5% out of the total). NOVA 4 prevailed in all the types and mostly in muesli (82%) and other cereals (95%), with the exception of puffed cereals in which 21 items (49%) were classified as NOVA 1 and 19 (44%) as NOVA 4.

Regarding nutrition claims (NCs), among the 232 products classified as NOVA 4, 78% of the items showed at least 1 NC, while within the 60 items in the NOVA 1 group, 60% showed an NC. Health claims were instead reported by 26% of items in the NOVA 4 group and in 10% of those in the NOVA 1 group.

Fibre-related NCs were similarly distributed in NOVA 1 and NOVA 4 groups, with 47% and 49% of products, respectively, carrying this type of claim. Conversely, NCs related to minerals and/or vitamins were mainly present in the NOVA 4 group, with 93% of the items showing this type, while only 7% of items within the NOVA 1 group had this NC. Other NCs were displayed in a few items across the NOVA groups.

Regarding the presence of wholegrain ingredients, 58% and 87% of items made with refined ingredients or only partially with wholegrain ingredients (i.e., at least one) were in the NOVA 4 group, respectively. Conversely, items made with wholegrains were almost equally present in the NOVA 1 (39%), NOVA 3 (28%), and NOVA 4 (33) groups.

The nutritional quality of breakfast cereals belonging to the three NOVA groups stratified by cereal types is reported in Table 2. By considering all the retrieved breakfast cereals, NOVA 4 products showed the highest energy and sugar content per 100 g, while NOVA 1 products were characterized by the highest content of fibre and protein and the lowest amount of sugars and salt. Intriguingly, NOVA 1 and NOVA 4 cereals showed higher total and saturated fats and lower carbohydrate amounts than NOVA 3 products. Concerning the different types of cereals, a high variability in results of the nutritional characteristics of products grouped for NOVA classification was found. In fact, bran cereals and other cereals do not show any difference for energy and saturate contents within the different NOVA groups, while for all the other products NOVA 4 is almost always higher in energy, total carbohydrates, sugar, and salt than NOVA 1 and, in some cases, also NOVA 3 products. When salt is taken into consideration, almost all the NOVA 3 and NOVA 4 groups contain a consistent higher amount of salt—up to ten to twenty times more—than NOVA 1 products. Except for muesli cereals, all the other products labelled as NOVA 1 resulted significantly higher in protein content than NOVA 4 and in most cases also compared to NOVA 3.

Nutritional information of products belonging to the different NOVA groups and stratified on the basis of whole grain ingredients is shown in Supplementary Table S1. Data evidenced a few differences, only in terms of sugar and salt contents, among whole grain products belonging to different NOVA groups. Concerning products made with refined grains and partially produced with wholegrain, data evidenced significantly lower contents of energy, carbohydrate, sugar, fibre, and protein in NOVA 1 products compared to the NOVA 3 and 4 ones. It is worth underlining that the three product categories (refined grain, partially produced with wholegrain, and wholegrain products) have a different number of items, which may impact the intra-product variability of the data (Table 1). Data were also grouped on the basis of the tertiles of sugar, fibre, and salt amounts and then compared on the basis of the NOVA group (Supplementary Table S2). Concerning tertilization based on the sugar amount, products did not reveal particular differences between the NOVA groups, except for NOVA 1 products in the first tertile, which were lower in energy, total and saturated fats, carbohydrates, sugars, and salt and higher in fibre and protein compared to NOVA 2 and 3 products. When products were stratified for tertiles of fibre content, a generally worse nutritional profile in NOVA 4 compared to NOVA 1 items was observed, but here, again, the different number of items should be carefully considered. Finally, the salt tertilization did not show any result worth being highlighted, except for sugar amounts, which were much higher in NOVA 3 and NOVA 4 compared to NOVA 1 group.

### 3.2. Nutri-Score and NutrInform Battery of Breakfast Cereals

When only the Nutri-Score of the products was taken into consideration, regardless of the technological process, data showed that 141 (40%) items were labelled as C, 105 (30%) as A, 58 (17%) as D, and the remaining 45 (13%) items had a Nutri-Score B.

**Figure 1:**
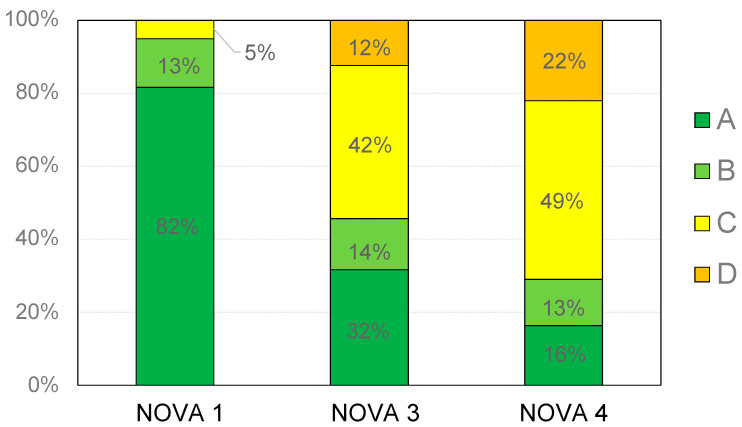
Percentage distribution of breakfast cereals based on NOVA group and Nutri-Score.

Figure 1 reports the distribution of the retrieved breakfast cereals according to NOVA categorization and Nutri-Score. As shown, NOVA 1 breakfast cereals displayed the highest proportion of products with Nutri-Score A (*n* = 49; 82%), followed by products belonging to NOVA 3 (*n* = 18; 32%) and NOVA 4 (*n* = 38; 16%) groups. The NOVA 1 group also showed the lowest proportion of products with Nutri-Score C (*n* = 3; 5%). Conversely, the Nutri-Score C prevailed in both NOVA 3 (*n* = 24; 42%) and NOVA 4 (*n* = 114; 49%) groups.

Finally, no products with Nutri-Score D were found in the NOVA 1 group, while 7 items (12%) and 51 items (22%) with Nutri-Score D were found in the NOVA 3 and NOVA 4 groups, respectively. No products displayed a Nutri-Score E.

**Figure 2:**
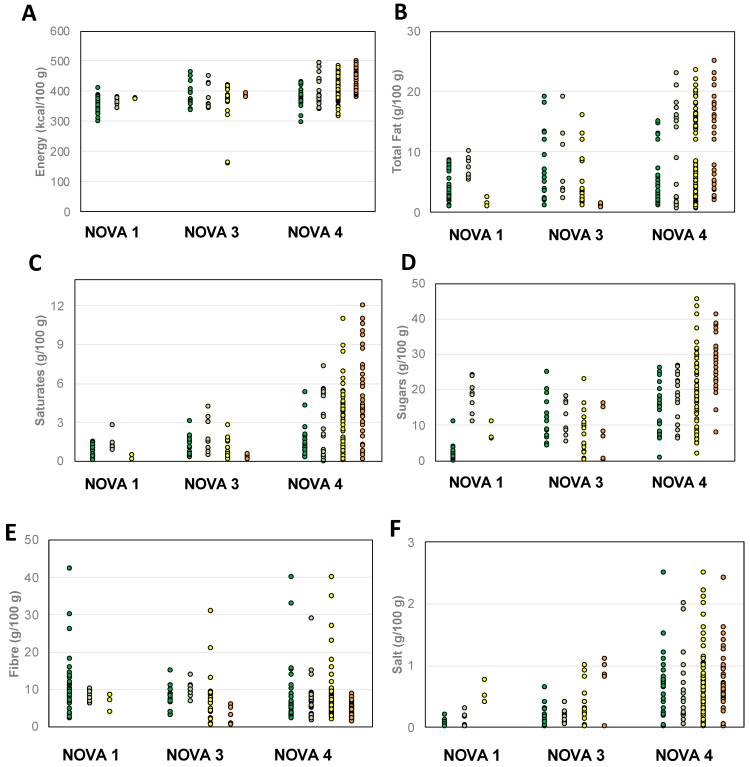
Energy (**A**) and nutrient (**B**–**F**) content of breakfast cereals grouped on the basis of NOVA group and Nutri-Score. Legend of Nutri-Scores: 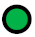 = A; 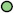 = B; 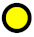 = C; 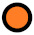 = D.

Figure 2 reports the distribution of energy and some nutrient contents in breakfast cereals, classified according to the NOVA group and Nutri-Score. On the whole, a wide variability in terms of energy, nutrients, and fibre has been found regardless of the NOVA group and Nutri-Score values. Almost all the products, irrespective of the Nutri-Score value and the NOVA group, fell within the range of energy 300–500 kcal/100 g; data showed that NOVA 3 and NOVA 4 products had the largest variability in terms of total and saturated fats, sugar, and salt, with no exceptions among the Nutri-Score values. NOVA 1 products showed lower values of total and saturated fats than NOVA 3 and 4 ones, with some exceptions. Concerning fibre values, only Nutri-Score D products had lower than 10 g/100 g fibre content; all the other products showed a wide range of values, thus not allowing a clear grouping of products on the basis of their NOVA group or Nutri-Score.

**Figure 3:**
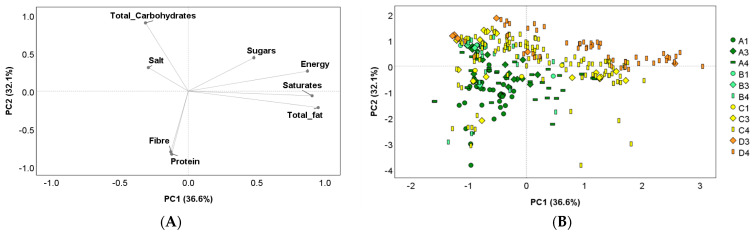
Principal component (PC) analysis describing the intra-group variability of products based on their nutrient composition (energy (kcal/100 g), total fat (g/100 g), saturates (g/100 g), total carbohydrates (g/100 g), sugars (g/100 g), protein (g/100 g), fibre (g/100 g), and salt (g/100 g)). Loading plots (**A**) of PC1 versus PC2; score plots (**B**) of the nutrient composition of each product analyzed organized according to Nutri-Score. Legend: legend is composed of letters (A to D) indicating the letters of the Nutri-Score and numbers (1, 3, and 4) indicating the NOVA groups.

The variability in the nutritional composition of the breakfast cereals was explored by PC analysis (Figure 3). Two PCs explained 68.7% of the total variability: (i) PC1—which accounted for 36.6% of the variability—was mainly positively loaded by energy, total and saturated fats, and sugars, while being negatively loaded by total carbohydrates, salt, fibre, and protein; (ii) PC2—explaining the 32.1% of the total variability—was mainly positively loaded by total carbohydrates, sugars, and salt and negatively loaded by fibre and protein (Figure 3A). The score plot in Figure 3B confirms a high variability that did not allow products to be grouped on the basis of the NOVA and Nutri-Score values. On the whole, most of the products with B, C, and D Nutri-Scores were described as having high energy, total carbohydrates, salt, total and saturated fats, and sugars, with no distinctions for NOVA groups. Then, a main characterization of the A products by fibre and protein was slightly evidenced, but no distinction among NOVA groups could be pointed out.

Regarding the NutrInform battery, Table 3 reports the batteries of products classified based on the NOVA groups and grouped for the breakfast cereal typologies. Data confirmed the evidence of a tight variability for daily energy contribution of a 30 g serving of the products, from 5 to 7%, with few distinctions among the NOVA groups. On the contrary, most of the variability referred to the contribution to the daily amounts of total and saturated fats, but just for a few typologies. Interestingly, NOVA 3 “other cereals” accounted for up to 14% of daily saturated fats vs. 3% of the NOVA 4; nevertheless, it is worth to remember that, among “other cereals”, only three NOVA 3 items were retrieved compared to fifty-six NOVA 4. On the whole, NOVA 1 products were almost not contributing to the daily salt amount; the items with the most impact were bran cereals and flakes, both NOVA 3 and 4, with up to 8% of the daily salt amount. Finally, despite all the products not being largely different for the daily sugar amounts, NOVA 3 and NOVA 4 puffed cereals contributed to 8% and 11% of sugar daily amounts, respectively.

## 4. Discussion

The present manuscript analyzed the breakfast cereals currently on the Italian market in terms of both nutritional quality—intended as nutritional values retrieved in the food labelling and as some of the FOPNLs proposed so far (i.e., Nutri-Score and NutrInform battery)—and level of processing, according to the NOVA system developed by Monteiro and colleagues with the intention to classify foods into four groups based on the type of processing [12]. Considering the number and types of items retrieved, some general considerations can be made. First, compared with our previous survey conducted in 2019 [11], we found an increasing number of breakfast cereal products sold on the market; in particular, muesli increased from 54 to 104 and puffed cereals from 29 to 43 products, for a total of 349 retrieved products.

Regarding the nutritional quality, we found several differences between the categorization with the Nutri-Score and that with the NutrInform battery. Specifically, 70% of breakfast cereals were labelled as Nutri-Score C and A (40% and 30%, respectively), followed by D and B (17% and 13%, respectively), while no products scoring E were found. On the Spanish market, Morales et al. found similar percentages of B and C among 53 breakfast cereals sold in 2018, while fewer A-labelled products (19%) and more D-labelled products (30%) were observed compared to the present study [14]. Vermote and colleagues analyzed the distribution of Nutri-Score among breakfast cereals in the Belgian market, in order to compare changes between 2017 and 2018 [15]. In both years, the authors found a prevalence of Nutri-Score C (43.4% and 40.6% in 2017 and 2018, respectively), A (25.0% and 29.7%), and D (22.8% and 17.9%), while only 8.4% and 11.5% scored B and only one item scored E in both years.

When the NutrInform battery was used, the differences among products were lower since in this FOPNL the nutrient content of specific components is expressed considering the serving size of 30 g, as suggested by the Italian food-based dietary guidelines [16]. These discrepancies between Nutri-Score and NutrInform battery in evaluating the nutritional quality of those products further highlight the differences between these two types of FOPNLs in providing information about the nutritional quality of food products.

Regarding the level of processing, we classified the breakfast cereals in three out of the four groups based on the type of the food processing as described by the NOVA system [12]. A large majority of products were classified as NOVA 4, but we also found minimally processed items classified in the NOVA 1 group and others with added culinary ingredients (e.g., salt and sugar), thus falling in the NOVA 3 group. These results are in line with the ones found by Morales et al. [14] who reported that 59%, 30%, and 11% of products were labelled as NOVA 4, 3, and 1, respectively.

In this survey, we also aimed at understanding whether NOVA and Nutri-Score describe the nutritional quality of the food products in a similar way by considering breakfast cereals, a food group whose products belong to many NOVA and Nutri-Score groups. When the products were grouped according to NOVA classification, we found that most of the items belonging to the NOVA 4 group were characterized by C and D Nutri-Score letters, which implies a medium-to-low nutritional quality. However, if from one side we found no NOVA 1 products labelled as Nutri-Score D and more than 80% of them labelled as A, then from the other side we retrieved many B and C products as in NOVA 1 as well as in NOVA 3 and NOVA 4 items. These findings support the previous hypothesis that minimally processed foods show a better nutritional quality than processed and ultra-processed analogues, mainly attributable to lower amounts of added ingredients, i.e., sugar and salt [17]. Nevertheless, the presence of some C Nutri-Score products in the NOVA 1 group underlines the concept that nutritional quality and food processing are not always in agreement to describe food characteristics. Regarding the NutriInform battery and NOVA classification, by considering the single energy and nutrient contents per 30 g serving of breakfast cereals, data showed that, except for specific types (i.e., muesli) and nutrients (i.e., total fats), the change of the battery loads across the different NOVA groups was pretty tight. Consequently, these data point out that there is no absolute consensus that the lowest nutritional quality, described by means of Nutri-Score and NutrInform battery, can be totally ascribed to the technological process of the food. For this reason, we do believe that the consumer should be carefully taught how to read and understand all the information on the food pack for making conscious shopping choices, independently of the FOPLNs.

As to the relationship between NOVA and Nutri-Score, our findings are in line with those of a survey concerning almost 10,000 various products sold on the Spanish market, showing that NOVA 3 and NOVA 4 items are widely characterized by all kinds of Nutri-Score letters and the NOVA 1 group is poorly represented by C to E Nutri-Score items [10]. Similar results have been found also by a Chilean study performing a crossing-ranking analysis between Nutri-Score and NOVA FOPLNs of 736 food products sold on the Chilean market [18]. Data showed that (i) for the NOVA 4 products, 70% out of the total were labelled as Nutri-Score B; (ii) for the NOVA 3 products, 25% and 2% of the products were characterized by Nutri-Score A and E, respectively. Additionally, in this case, the majority of the NOVA 1 products fell in the classifications A and B of the Nutri-Score [18]. The wide variability of the nutritional quality—evaluated by means of the Nutri-Score—of more than 220,000 products classified as NOVA 4 sold in France in 2020 was well described in the paper of Galán et al. [19]. In fact, despite only 21% of the products being labelled as A or B, the remaining ones were almost equally characterized by a C, D, or E Nutri-Score, underlying the wide variability in terms of the nutritional quality of ultra-processed foods. The same authors also showed that, by considering 2,036 products used in the NutriNet-Santé study, 58% to 86% of the products were ultra-processed ones, independent of the Nutri-Score letter [19].

Therefore, we are here to argue whether both the NOVA and Nutri-Score systems may converge in a unique definition of the healthiness of the product. This uncertainty in defining the healthiness of breakfast cereals has been deeply considered by Dickie et al. [20], who analyzed—within the “cereal and cereal products” group—221 breakfast cereals present on the Australian market. The authors calculated a percentage value of agreement between the classification of “healthy” and “unhealthy” by different FOPLNs, among which were Nutri-Score and NOVA. The value for breakfast cereals was 23%, which has been classified as a “high degree of disagreement” between the classification of healthiness for the two FOPNLs [20]. Authors attributed these disagreements between the two FOPNL to the different aspects considered for the definition of the healthiness of the products: while Nutri-Score has a nutrient-based scheme for the calculation of the different values, NOVA does not profile nutrients, but just the processing. This means that, for example, the presence of high amounts of salt or sugars in breakfast cereals—as also confirmed by data from our group [11,21]—are differently taken into account by Nutri-Score and NOVA algorithms, i.e, low Nutri-Score and minimally processed items. On the contrary, the presence of industrial ingredients and additives, which classifies the product as “not healthy” by the NOVA system, is not considered for the Nutri-Score. However, these last characteristics of the NOVA classification—ingredient addition and healthiness of the product—should be carefully contemplated. An Australian study examined different breakfast models that include or do not include ultra-processed breakfast cereals, categorized according to fortification/addition with vitamins, minerals, or fibre [22]. The authors evaluated whether those dietary models met the nutrient requirement by the Australian Dietary Guidelines, which discourages the consumption of ultra-processed foods at the expense of minimally or not processed foods. Data showed that the exclusion of such ultra-processed foods—among which are breakfast cereals—resulted in a significantly lower intake of key nutrients, such as some vitamins and iodine, with potentially harmful health consequences [22].

One of the main future goals to pursue in the nutrition field is to educate the customer in reading and understanding the whole information present on the food pack and, particularly, the FOPLNs boasted on the products and their differences in depicting the nutritional quality. We demonstrate here, for example, that i) many items rated as A or B had similar energy, total fats, and sugars regardless of the NOVA group and ii) within each NOVA group, many products with different Nutri-Score values have a similar nutritional profile, especially for serving, highlighting that the technological process of the food cannot be a descriptor of its nutritional quality.

## 5. Conclusions

The presence of FOPLNs on food packs for several different food groups is globally increasing, with the intention to help the consumer to recognize the key nutritional and technological aspects of the food and invite him to consider the global healthiness of the product. If from one side this effort may result in a global improvement of the dietary habits of the citizens—as confirmed by many epidemiological surveys relating the better scores of Nutri-Score and NOVA products to the lowering of risks of obesity and chronic diseases—from another side, our data show that the agreement of the different FOPLNs in describing the whole healthiness of the product is not valid for all the food groups. This disagreement has been deeply discussed in this paper, mainly explained by the different characteristics of the food considered for the development of the Nutri-Score and Nutri-Inform battery (mainly energy, nutrients, and some ingredients) and NOVA system (mainly the degree of process of the food). Not least, the concept of “healthiness” should not only be attributed to a single food but also to the quantity and the frequency of consumption as well as the influence of such food on the whole diet of the single individual.

In conclusion, the findings of the present survey suggest that neither the NOVA system nor the Nutri-Score or NutriInform battery are capable to describe the healthiness of the breakfast cereal products in a similar way. The three different FOPLNs give information on different characteristics of the products. We here rebate that the simple presence of symbols or colors on the food pack cannot drive the intention-to-buy of the customers. On the contrary, they should be carefully trained in how to read and understand the nutritional information present on the food pack and in how to interpret the FOPLNs on the item, and they should be left with the choice of which breakfast cereals satisfy their nutritional and health needs.

## Figures and Tables

**Table 1:** Number and characteristics of retrieved breakfast cereals stratified based on the NOVA group.

		NOVA Groups		
		NOVA 1	NOVA 3	NOVA 4
Total		60	57	232
Type	Muesli	2	17	85
	Flakes	29	33	63
	Bran cereals	8	1	9
	Puffed cereals	21	3	19
	Other cereals	0	3	56
Organic	No	13	28	176
	Yes	47	29	56
Branded	No	17	30	118
	Yes	43	27	114
Nutrition claim	No	24	14	52
	Yes	36	43	180
Fibre claim	No	28	33	114
	Yes	32	24	118
Fat claim	No	54	42	207
	Yes	6	15	25
Salt claim	No	54	55	229
	Yes	6	2	3
Vitamin and mineral claim	No	56	42	126
	Yes	4	15	106
Sugar claim	No	50	51	218
	Yes	10	6	14
Protein claim	No	47	54	221
	Yes	13	3	11
Health claim	No	54	51	172
	Yes	6	6	60
	No	37	30	95
Wholegrain	Partially #	4	13	121
	Yes	19	14	16

Legend: NOVA 1: minimally processed foods; NOVA 3, processed foods; NOVA 4, ultra-processed foods. # Items partially produced with wholegrain ingredients (i.e., at least one).

**Table 2:** Energy, nutrients, and salt content of retrieved breakfast cereals stratified based on the NOVA group.

	NOVA	Energy (kJ/100 g)	Energy (kcal/100 g)	Total Fat (g/100 g)	Sfa (g/100 g)	Carbohydrates (g/100 g)	Sugar (g/100 g)	Fibre (g/100 g)	Protein (g/100 g)	SALT (g/100 g)
All	NOVA 1	1549 (1514–1596) c	366 (358–378) c	5.0 (2.6–7.0) a	0.9 (0.5–1.3) a	61.0 (57.7–69.0) b	1.1 (0.7–2.0) c	9.4 (6.5–12.1) a	12.1 (11.0–13.8) a	0.0 (0.0–0.0) B
	NOVA 3	1584 (1564–1634) b	375 (370–385) b	2.1 (1.0–7.3) b	0.5 (0.3–1.1) b	73.0 (60.7–82.0) a	15.0 (6.6–20.4) b	6.0 (3.0–8.8) b	8.5 (7.3–11.0) b	0.4 (0.2–1.0) A
	NOVA 4	1668 (1601–1837) a	396 (378–438) a	5.7 (2.6–15.0) a	1.6 (0.6–4.0) a	69.0 (61.0–78.8) b	20.0 (14.0 -25.0) a	6.0 (4.3–8.0) b	8.5 (7.4–10.0) b	0.5 (0.2–0.9) A
Muesli	NOVA 1	1499 (1467–1531) b	356 (348–364) a,b	6.6 (5.9–7.3) b	1.6 (1.0–2.1) a	58.5 (58.0–59.0) a	18.5 (11.0–26.0) a	11.0 (11.0–11.0) a	10.2 (9.3–11.0) a	0.03 (0.02–0.03) A,B
	NOVA 3	1756 (1565–1820) a,b	418 (372–434) b	12.7 (7.3–16.0) a	4.3 (1.1–5.3) a	59.0 (58.0–63.2) a	18.0 (16.0–22.0) a	8.0 (7.1–9.0) a,b	10.0 (9.3–11.0) a	0.2 (0.02–0.2) B
	NOVA 4	1834 (1746–1917) a	437 (416–458) a	16.0 (13.0–19.0) a	4.1 (1.9–5.1) a	61.1 (58.0–63.2) a	20.0 (16.0–24.0) a	7.2 (6.2–8.4) b	9.0 (8.5–11.0) a	0.2 (0.1–0.4) A
Flakes	NOVA 1	1549 (1517–1566) b	366 (360–372) b	7.0 (5.4–7.0) a	1.2 (1.0–1.3) a	59.1 (58.6–63.0) b	1.1 (0.7–1.4) b	9.9 (8.0–10.0) a	12.0 (11.6–13.0) a	0.01 (0.01–0.03) B
	NOVA 3	1578 (1564–1604) a	372 (370–378) a	1.0 (1.0–1.6) c	0.3 (0.2–0.5) b	81.0 (81.0–83.0) a	8.0 (6.2–16.0) a	3.3 (3.0–6.6) b	8.0 (7.3–8.5) b	0.9 (0.5–1.9) A
	NOVA 4	1605 (1574–1662) a	379 (371–390) a	2.0 (1.5–5.0) b	0.5 (0.3–1.5) b	78.0 (73.0–81.0) a	14.0 (7.8–17.8) a	4.7 (63.5–6.3) b	8.1 (7.4–11.6) b	0.8 (0.5–1.4) A
Bran cereals	NOVA 1	1412 (1291–1526) a	336 (309–363) a	6.1 (4.5–7.5) a	1.1 (0.8–1.3) a	47.5 (37.0–52.1) a	2.0 (1.2–2.7) b	22.1 (16.0–30.0) c	14.6 (13.5–15.4) a	0.01 (0.002–0.01) B
	NOVA 3	1436 (1436–1436) a	343 (343–343) a	4.5 (4.5–4.5) a,b	0.9 (0.9–0.9) a	46.0 (46.0–46.0) a	18.0 (18.0–18.0) a	29.0 (29.0–29.0) b	15.0 (15.0–15.0) a,b	1.2 (1.2–1.2) A
	NOVA 4	1342 (1318–1342) a	321 (316–321) a	3.9 (3.5–3.9) b	0.7 (0.7–1.0) a	41.0 (38.0–41.0) a	17.0 (13.0–18.0) a	35.0 (33.0–35.0) a	14.0 (13.0–14.6) b	1.3 (1.0–1.3) A
Puffed cereals	NOVA 1	1600 (1541–1611) b	378 (365–381) b	2.5 (1.1–3.1) a	0.5 (0.4–0.6) b	71.0 (68.0–85.0) b	0.6 (0.5–1.7) b	6.8 (0.8–8.5) a	11.5 (7.1–14.0) a	0.01 (0.003–0.01) B
	NOVA 3	1620 (1570–1673) a,b	382 (375–396) a,b	4.2 (1.5–5.7) a	0.7 (0.5–0.8) a	75.8 (73.0–85.0) a,b	25.0 (22.0–45.4) a	2.8 (2.2–7.5) a	9.6 (6.0–12.6) b	0.02 (0.000–0.3) A
	NOVA 4	1651 (1609–1690) a	390 (385–400) b	3.2 (1.9–5.1) a	0.6 (0.5–1.0) a	79.0 (78.0–85.0) a	33.0 (23.0–41.0) a	4.6 (2.5–5.5) a	7.0 (5.5–8.5) b	0.1 (0.01–0.7) A
Other cereals	NOVA 1	-	-	-	-	-	-	-	-	-
	NOVA 3	1992 (1531–2016)	476 (362–481)	22.0 (2.0–22.0)	8.9 (0.6–11.0)	60.0 (60.0–69.0)	19.0 (4.2–27.0)	5.7 (4.5–10.0)	8.0 (7.2–12.0)	0.6 (0.3–0.9)
	NOVA 4	1652 (1617–1864)	390 (382–443)	4.6 (2.8–14.1)	1.8 (0.9–4.0)	73.9 (69.4–79.0) *	25.0 (22.7–29.0)	5.0 (3.9–6.8)	7.7 (6.7–8.7)	0.7 (0.5–0.9)

Legend: For each group, different letters or asterisks in the same column after parenthesis indicate significant differences among types (Kruskal–Wallis test for independent samples with multiple pairwise comparisons or Mann–Whitney non-parametric test for two independent samples, *p* < 0.05). SFA, saturates.

**Table 3:** NutrInform battery of breakfast cereals stratified on the basis of the NOVA group.

	NOVA 1	NOVA3	NOVA 4
All	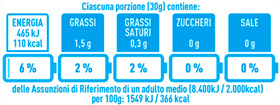	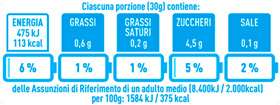	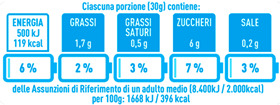
*Muesli*	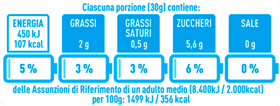	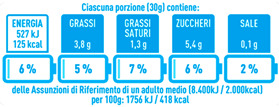	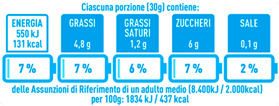
*Flakes*	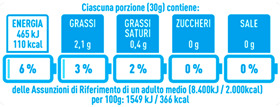	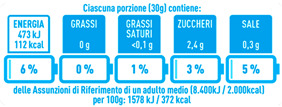	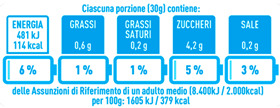
*Bran Cereals*	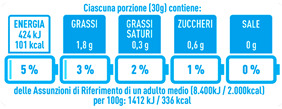	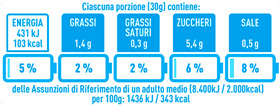	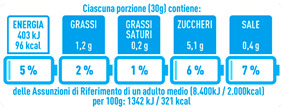
*Puffed Cereals*	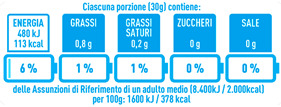	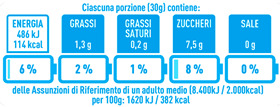	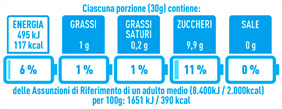
*Other Cereals*	No items	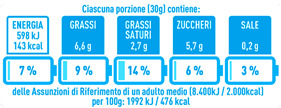	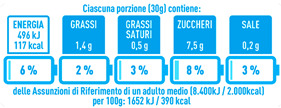

## Data Availability

The data presented in this study are available on request from the corresponding author.

## Supplementary Materials

The following supporting information can be downloaded at: https://www.mdpi.com/article/10.3390/nu15082013/s1, Table S1: energy, nutrients, and salt content of retrieved breakfast cereals, stratified based on the presence of whole grain ingredients and the NOVA group; Table S2: energy, nutrients, and salt content of retrieved breakfast cereals, stratified based on tertiles of sugar, fibre, and salt content and the NOVA group.

## Appendix A

**Table A1:** SINU Young Working Group.

Margherita Dall’Asta	Department of Animal Science, Food and Nutrition, Università Cattolica del Sacro Cuore, Piacenza, Italy
Gaetana Paolella	Department of Chemistry and Biology “A. Zambelli”, University of Salerno, Fisciano, Italy
Marika Dello Russo	Institute of Food Sciences, National Research Council, Avellino, Italy
Stefania Moccia	Institute of Food Sciences, National Research Council, Avellino, Italy
Carmela Spagnuolo	Institute of Food Sciences, National Research Council, Avellino, Italy
Daniele Nucci	Veneto Institute of Oncology IOV-IRCCS, Padova, Italy
Veronica Pignone	Freelance Nutritionist, San Giorgio del Sannio, Italy
Emilia Ruggiero	Department of Epidemiology and Prevention, IRCCS Neuromed, Pozzilli, Italy
Alice Rosi	Department of Food and Drug, University of Parma, Italy
Giorgia Vici	School of Biosciences and Veterinary Medicine, University of Camerino, Italy
