# Supplementary material for: Processing and Nutritional Quality of Breakfast Cereals Sold in Italy: Results from the Food Labelling of Italian Products (FLIP) Study
Source: Nutrients. 2023 Apr 21;15(8):2013. doi: 10.3390/nu15082013 (PMC10145012; doi:10.3390/nu15082013)
Supplement: Supplementary file 1 [file nutrients-15-02013-s001.zip › nutrients-2296825-supplementary.pdf]

**Supplementary Table S1.** Energy, nutrients and salt content of retrieved breakfast cereals, stratified based on the presence of whole grain ingredients and the NOVA group.

| | NOVA | Energy (kJ/100 g) | Energy (kcal/100 g) | Total Fat (g/100 g) | Sfa (g/100 g) | Carbohydrates (g/100 g) | Sugar (g/100 g) | Fibre (g/100 g) | Protein (g/100 g) | Salt (g/100 g) |
|---|---|---|---|---|---|---|---|---|---|---|
| RG | NOVA 1 | 1555 (1523-1601)b | 369 (360-378)b | 3.1 (2.0-7.0)a | 0.6 (0.4-1.2)b | 64.3 (59.0-74.0)b | 0.9 (0.5-1.4)c | 8.4 (3.7-10.0)a | 11.8 (9.2-13.0)a | 0.0 (0.0-0.0)b |
| | NOVA 3 | 1593 (1570-1620)b | 375 (371-382)b | 1.0 (1.0-1.6)b | 0.3 (0.2-0.8)b | 81.0 (81.0-83.4)a | 8.3 (6.1-22.0)b | 3.2 (2.8-4.0)b | 8.0 (7.0-8.5)b | 0.8 (0.3-1.9)a |
| | NOVA 4 | 1664 (1609-1851)a | 393 (380-441)a | 5.1 (2.5-15.0)a | 1.6 (0.6-4.2)a | 71.0 (62.0-81.0)b | 22.0 (10.0-27.0)a | 4.9 (3.2-7.7)b | 8.0 (6.9-9.9)b | 0.5 (0.1-1.0)a |
| PWG | NOVA 1 | 1317 (1291-1429)b | 315 (309-340)b | 4.5 (3.8-5.0)a | 0.8 (0.7-0.8)a | 41.5 (37.0-53.5)b | 2.3 (1.5-2.7)b | 28.0 (17.6-30.0)a | 14.5 (13.0-15.0)a | 0.0 (0.0-0.0)b |
| | NOVA 3 | 1648 (1580-1829)a | 390 (376-436)a | 7.3 (2.5-16.0)a | 1.0 (0.6-5.5)a | 60.0 (58.6-75.0)ab | 18.0 (15.0-20.4)a | 8.5 (5.7-9.6)ab | 8.7 (8.0-10.0)b | 0.3 (0.2-0.8)a |
| | NOVA 4 | 1685 (1596-1834)a | 399 (377-442)a | 6.2 (2.8-15.8)a | 1.8 (0.6-3.9)a | 68.0 (61.0-76.0)a | 19.0 (15.0-24.8)a | 6.4 (5.2-8.0)b | 8.5 (7.9-10.8)b | 0.5 (0.3-0.8)a |
| WG | NOVA 1 | 1549 (1516-1592)a | 368 (359-376)a | 7.0 (3.6-7.7)a | 1.2 (0.6-1.3)a | 59.0 (56.0-66.0)a | 1.3 (1.0-2.0)b | 10.0 (7.1-14.4)a | 13.0 (12.0-14.1)a | 0.0 (0.0-0.0)b |
| | NOVA 3 | 1551 (1522-1584)a | 370 (361-375)a | 3.4 (2.2-6.2)a | 0.8 (0.5-1.2)a | 65.0 (61.0-70.0)a | 16.0 (9.9-18.9)a | 8.2 (7.0-14.0)a | 11.5 (11.0-13.0)a | 0.3 (0.0-0.8)a |
| | NOVA 4 | 1603 (1431-1704)a | 379 (341-405)a | 4.6 (2.9-10.7)a | 0.9 (0.7-2.3)a | 59.2 (40.1-70.0)a | 15.0 (10.0-21.5)a | 8.2 (5.5-14.9)a | 11.8 (8.7-14.5)a | 0.2 (0.1-1.0)a |

Legend: For each group, different letters in the same column after parenthesis indicate significant differences among types (Kruskal–Wallis test for independent samples with multiple pairwise comparisons, $p < 0.05$). RG: refined grain; PWG: partially produced with wholegrain; WG: wholegrain; SFA, saturates.

**Supplementary Table S2.** Energy, nutrients and salt content of retrieved breakfast cereals, stratified based on tertiles of sugar, fibre and salt content and the NOVA group.

| | | **NOVA** | **Energy (kJ/100 g)** | **Energy (kcal/100 g)** | **Total Fat (g/100 g)** | **Sfa (g/100 g)** | **Carbohydrates (g/100 g)** | **Sugar (g/100 g)** | **Fibre (g/100 g)** | **Protein (g/100 g)** | **Salt (g/100 g)** |
|---|---|---|---|---|---|---|---|---|---|---|---|
| SUGAR | Tertile 1 | NOVA 1 | 1549 (1514-1592)b | 366 (358-377)b | 4.3 (2.5-7.0)a | 0.8 (0.5-1.3)a | 61.3 (57.4-70.5)b | 1.1 (0.7-1.5)b | 9.4 (6.5-11.3)a | 12.6 (11.0-14.0)a | 0.0 (0.0-0.0)b |
| | | NOVA 3 | 1574 (1570-1597)a | 371 (370-377)a | 1.0 (1.0-1.2)b | 0.3 (0.2-0.3)b | 81.0 (81.0-82.0)a | 6.5 (6.0-6.6)a | 3.3 (3.0-4.0)b | 7.9 (7.3-8.0)b | 1.0 (0.4-2.0)a |
| | | NOVA 4 | 1605 (1536-1658)a | 379 (364-392)a | 2.6 (1.5-9.1)b | 0.6 (0.4-1.7)a | 71.0 (58.5-81.0)a | 7.3 (5.7-8.8)a | 6.6 (4.0-10.5)a | 10.0 (8.0-12.0)a,b | 0.8 (0.1-1.4)a |
| | Tertile 2 | NOVA 1 | 1611 (1531-1691)a | 383 (364-402)a | 9.7 (7.3-12.0)a | 1.9 (1.0-2.8)a | 59.5 (58.0-61.0)a | 11.5 (11.0-12.0)a | 7.7 (4.3-11.0)a | 11.0 (11.0-11.0)a | 0.0 (0.0-0.0)a |
| | | NOVA 3 | 1589 (1526-1792)a | 377 (362-426)a | 5.8 (2.1-12.7)a | 1.0 (0.5-4.3)a | 62.0 (58.6-75.0)a | 17.0 (12.0-18.2)a | 8.5 (6.0-10.1)a | 10.0 (8.0-11.0)a | 0.3 (0.0-0.8)a |
| | | NOVA 4 | 1662 (1573-1839)a | 393 (371-441)a | 6.1 (2.1-16.4)a | 1.5 (0.6-4.0)a | 64.0 (57.1-76.0)a | 17.0 (14.4-19.0)a | 6.9 (5.3-8.5)a | 9.4 (8.1-12.0)a | 0.5 (0.2-0.8)a |
| | Tertile 3 | NOVA 1 | 1623 (1467-1779)a | 386 (348-423)a | 9.0 (5.9-12.0)a | 1.9 (1.7-2.1)a | 62.5 (59.0-66.0)a | 25.0 (24.0-26.0)a | 9.0 (6.9-11.0)a | 9.5 (9.3-9.6)a | 0.0 (0.0-0.0)a |
| | | NOVA 3 | 1614 (1570-1756)a | 381 (375-418)a | 5.3 (1.5-15.0)a | 0.8 (0.5-5.3)a | 65.0 (60.0-85.0)a | 24.0 (22.0-26.5)a | 5.0 (2.6-7.5)a | 8.5 (6.0-9.6)a | 0.2 (0.2-0.5)a |
| | | NOVA 4 | 1698 (1637-1889)a | 402 (386-449)a | 7.0 (3.5-15.3)a | 2.3 (1.0-4.5)a | 72.0 (63.0-79.0)a | 26.0 (24.1-29.7)a | 5.4 (3.9-6.7)a | 8.0 (7.0-8.6)a | 0.5 (0.2-0.8)a |
| FIBRE | Tertile 1 | NOVA 1 | 1611 (1592-1618)a,b | 380 (376-381)a,b | 1.1 (1.1-4.0)a,b | 0.4 (0.4-0.5)a,b | 82.5 (74.0-85.0)a,b | 0.5 (0.2-1.1)c | 2.2 (0.5-3.7)b | 7.2 (7.1-9.2)a | 0.0 (0.0-0.0)b |
| | | NOVA 3 | 1591 (1574-1605)b | 375 (371-378)b | 1.0 (0.9-1.2)b | 0.3 (0.2-0.4)a | 82.0 (81.0-84.0)a | 6.6 (6.0-22.0)b | 3.0 (2.6-3.3)b | 7.3 (7.0-8.0)a | 1.0 (0.5-2.0)a |
| | | NOVA 4 | 1633 (1602-1705)a | 385 (378-403)a | 2.6 (1.8-5.6)a | 0.7 (0.4-2.3)a | 79.0 (72.0-82.0)b | 22.0 (11.0-27.0)a | 3.8 (3.0-4.3)a | 7.3 (6.4-9.0)a | 0.9 (0.5-1.1)a |
| | Tertile 2 | NOVA 1 | 1578 (1528-1601)b | 374 (361-378)b | 3.1 (2.6-5.6)b | 0.6 (0.5-1.1)b | 67.5 (64.5-70.5)a | 1.8 (1.2-2.4)b | 7.0 (6.9-7.1)a | 14.0 (11.5-14.5)a | 0.0 (0.0-0.0)b |
| | | NOVA 3 | 1632 (1565-1792)a,b | 385 (373-426)a,b | 7.6 (2.3-15.0)a | 1.0 (0.6-5.3)a,b | 63.8 (60.5-73.0)a | 16.1 (10.1-23.7)a | 6.9 (6.0-7.5)a | 10.3 (8.2-11.0)a,b | 0.2 (0.0-0.8)a |
| | | NOVA 4 | 1754 (1641-1890)a | 418 (386-450)a | 12.5 (4.1-17.0)a | 2.8 (1.0-4.5)a | 66.0 (61.0-75.9)a | 22.0 (18.0-26.0)a | 6.3 (5.6-6.8)a | 8.5 (8.0-9.4)b | 0.4 (0.1-0.7)a |
| | Tertile 3 | NOVA 1 | 1528 (1478-1554)b | 363 (348-369)b | 7.0 (3.1-7.0)a | 1.2 (0.8-1.3)b | 59.0 (57.0-61.0)a | 1.1 (0.7-1.9)b | 10.1 (9.5-13.0)a | 13.0 (11.8-14.0)a | 0.0 (0.0-0.0)b |
| | | NOVA 3 | 1538 (1515-1812)a,b | 364 (360-432)a,b | 5.7 (2.4-16)a | 1.0 (0.6-5.2)a,b | 60.4 (58.0-65.0)a | 17.5 (11.0-18.9)a | 9.8 (8.9-14.0)a | 10.0 (9.6-12.0)b | 0.2 (0.0-0.8)a |
| | | NOVA 4 | 1646 (1490-1794)a | 391 (353-428)a | 7.8 (3.8-15.5)a | 1.7 (0.7-3.8)a | 60.7 (51.0-65.0)a | 16.0 (10.0-19.0)a | 9.4 (8.4-14.0)a | 11.0 (9.0-13.0)b | 0.4 (0.1-0.7)a |
| SALT | Tertile 1 | NOVA 1 | 1545 (1514-1601)b | 367 (358-378)b | 5.0 (2.6-7.0)b | 0.9 (0.5-1.3)b | 60.7 (57.4-69.0)a | 1.1 (0.7-2.0)b | 9.5 (6.9-11.3)a | 12.0 (11.0-14.0)a | 0.0 (0.0-0.0)b |
| | | NOVA 3 | 1565 (1515-1673)b | 372 (360-396)b | 6.4 (5.6-8.9)a,b | 1.1 (0.9-2.8)a,b | 60.7 (58.6-64.0)a | 20.0 (11.0-24.0)a | 7.5 (6.9-9.6)a,b | 10.1 (9.6-12.3)a,b | 0.0 (0.0-0.0)b |
| | | NOVA 4 | 1709 (1636-1884)a | 407 (389-450)a | 12.5 (5.0-18)a | 2.5 (1.4-4.5)a | 62.5 (58.0-72.0)a | 18.4 (13.1-26.0)a | 7.0 (6.0-8.4)b | 9.9 (8.5-11.0)b | 0.1 (0.0-0.1)a |
| | Tertile 2 | NOVA 1 | 1522 (1478-1566)a | 367 (349-372)a | 4.8 (2.6-7.0)a | 0.8 (0.2-1.3)a | 61.6 (59.1-64.0)a | 1.1 (1.0-1.1)b | 9.3 (8.4-10.2)a | 13.2 (13.0-13.3)a | 0.2 (0.2-0.2)a,b |
| | | NOVA 3 | 1614 (1570-1820)a | 381 (372-434)a | 2.2 (1.4-16.0)a | 0.6 (0.3-5.3)a | 66.0 (59.0-81.0)a | 17.0 (6.5-22.0)a | 7.6 (4.0-8.9)a | 8.7 (7.2-10.0)b | 0.3 (0.2-0.4)b |
| | | NOVA 4 | 1758 (1625-1882)a | 419 (385-449)a | 13.0 (3.8-17.0)a | 2.5 (1.0-4.5)a | 64.1 (61.0-76.0)a | 22.0 (16.3-26.0)a | 6.2 (5.1-8.0)a | 8.2 (7.0-8.9)b | 0.4 (0.3-0.5)a |
| | Tertile 3 | NOVA 1 | 1555 (1554-1555)a | 366 (366-366)a | 4.1 (1.0-7.1)a | 0.8 (0.2-1.4)a | 72.0 63.0-81.0)b | 0.7 (0.5-0.8)b | 3.5 (2.0-5.0)a | 10.0 (7.3-12.6)a | 1.8 (1.0-2.5)a |
| | | NOVA 3 | 1589 (1570-1604)a | 375 (370-378)a | 1.2 (1.0-2.1)b | 0.3 (0.2-0.5)b | 81.0 (75.0-83.0)a | 8.5 (6.1-16.1)a | 3.3 (3.0-6.0)a | 8.0 (7.3-8.5)a | 1.0 (0.9-2.0)a |
| | | NOVA 4 | 1613 (1566-1665)a | 380 (370-393)a | 2.8 (1.8-5.8)b | 0.7 (0.4-2.1)a | 75.0 (69.0-81.0)b | 18.0 (10.9-25.0)a,b | 4.8 (3.5-6.9)a | 8.3 (7.3-11)a | 1.0 (0.8-1.3)a |

Legend: For each group, different letters in the same column after parenthesis indicate significant differences among types (Kruskal–Wallis test for independent samples with multiple pairwise comparisons, $p < 0.05$). SFA, saturates.
